# Serum Zonulin Levels as an Early Biomarker in Predicting the Severity and Complications of Acute Pancreatitis

**DOI:** 10.5152/eurasianjmed.2022.0272

**Published:** 2023-02-01

**Authors:** Ufuk Avcıoğlu, Hasan Eruzun

**Affiliations:** 1 Department of Gastroenterology, Faculty of Medicine, Ondokuz Mayıs University, Samsun, Turkey

**Keywords:** Acute pancreatitis, complications, zonulin, bacterial translocation

## Abstract

**Objective:**

Acute pancreatitis can cause local or systemic complications and has high morbidity and mortality rates. In the early stages of pancreatitis, a decrease in the barrier function of the intestines and an increase in bacterial translocation are observed. Zonulin is a marker used to evaluate the integrity of the intestinal mucosal barrier. We aimed to investigate whether measuring serum zonulin levels would contribute to the early prediction of complications and severity in acute pancreatitis.

**Materials and Methods:**

Our study was an observational, prospective study and included 58 patients with acute pancreatitis and 21 healthy controls. Causes of pancreatitis and serum zonulin levels of the patients at the time they were diagnosed with pancreatitis were recorded. The patients were evaluated in terms of pancreatitis severity, organ dysfunction, complications, sepsis, morbidity, length of hospital stay, and mortality

**Results:**

Zonulin levels were higher in the control group and lowest in the severe pancreatitis group. No ­significant difference was observed in zonulin levels according to disease severity. There was no significant difference between zonulin levels in patients who developed organ dysfunction or sepsis. In patients with acute pancreatitis complications, zonulin levels were found to be significantly lower with a mean of 8.6 ng/mL (*P* < .02).

**Conclusion:**

Zonulin levels are not a guide in the diagnosis of acute pancreatitis, in determining its severity, and in the development of sepsis and organ dysfunction. The zonulin level at the time of diagnosis may be helpful in predicting complicated acute pancreatitis. Zonulin levels are not effective in demonstrating necrosis or infected necrosis.

**Main Points**

- Bacterial translocation, infected necrosis, and development of sepsis are associated with poor prognosis in acute pancreatitis.
- Zonulin is a tight junction protein that reflects intestinal permeability associated with bacterial translocation.
- The relationship between the development of sepsis, infected necrosis, and other complications in acute pancreatitis and the zonulin levels at the time of diagnosis was investigated.
- In acute pancreatitis, zonulin levels at the time of diagnosis were found to be significantly lower in patients who developed complications during follow-up.

## Introduction

Acute pancreatitis (AP) is a serious inflammation of the pancreas, manifested by abdominal pain and elevated pancreatic enzymes. It can cause local or systemic complications and has high morbidity and mortality rates. It is one of the most common causes of hospitalization originating from the gastrointestinal system and constitutes an important workload for health systems.^1^ The diagnosis and morbidity of pancreatitis have increased in the last 2 decades, with increased incidence of obesity, gallstones, and alcoholism.^1,2^ Premature activation of digestive enzymes has historically been recognized as the main mechanism of pancreatitis. The pathophysiology of AP consists of quite complex processes. It is clear that inappropriate activation of trypsinogen is responsible for acinar cell death and the early stage of AP.^3^ In addition, many factors from nuclear factor kappa B activation to trypsinogen gene mutation play a role in the pathophysiology of pancreatitis.^3^ The clinical spectrum of AP varies from mild pancreatic edema to infected pancreatic necrosis and even organ failure.^4^ In the current data, it has been shown that infected pancreatic necrosis determines the severity of the disease at least as much as organ failure and is closely related to mortality.^5^ In the early stages of pancreatitis, a decrease in the barrier function of the intestines and an increase in bacterial translocation are observed.^6,7^ Intestinal barrier dysfunction leading to bacterial translocation is responsible for pancreatitis, systemic inflammation, and sepsis.^8^ Many mechanisms have been discussed in the development of intestinal barrier dysfunction in AP. The resulting oxidative stress destroys the mucus layer and causes intestinal ischemia, epithelial cell, and mitochondrial damage that end up with the opening of tight and adherens junctions.^9^ The structure, function, and regulation of the intestinal epithelial barrier are provided by tight junctions.^10^ These connections regulate paracellular intestinal permeability. Tight junctions themselves are regulated by more than 50 proteins. One of them, zonulin, is the eukaryotic counterpart of the zonula occludens toxin of *Vibrio cholera*.^11^ Human zonulin (47 kDa protein) binds the epidermal growth factor receptor (EGFR) receptor and protease-activating receptor 2 in the intestinal epithelium. This complex disrupts the structure of the tight junctions of the small intestine by phosphorylating the zonula occludens proteins and increases intestinal permeability.^10^ Zonulin is the only measurable blood protein that reflects intestinal permeability.^11,12^ Although zonulin is mostly secreted from the liver, it can also be secreted from enterocytes, adipose tissue, brain, heart, immune system cells, lung, and kidney.^13^ High serum zonulin levels can be seen in various autoimmune diseases such as celiac disease and type 1 diabetes as well as in type 2 diabetes and obesity.^14^ Based on this information, we aimed to investigate whether measuring serum zonulin levels would contribute to the early prediction of intestinal barrier dysfunction and bacterial translocation in AP. We also tried to evaluate the utility of zonulin as a biomarker related to the clinical course and severity of the disease, development of necrosis, sepsis, and organ dysfunction in the early stages of pancreatitis.

## Materials and Methods

### Study Design

This study was planned as a prospective observational study. An informed consent form was obtained from all participants. The study was designed in accordance with the Declaration of Helsinki and approved by the ethics committee of Ondokuz Mayıs University (decision no: 2021/117; date: 15.03.2021).

### Patients

A total of 60 patients admitted to the emergency department of Ondokuz Mayıs University Hospital, Samsun, Turkey and diagnosed with AP according to the revised Atlanta criteria were included in the study. During follow-up, 1 patient was diagnosed with Crohn’s disease and 1 patient was diagnosed with lymphoma, so they were excluded from the study. The remaining 58 patients were enrolled into the study. Amylase and lipase levels were observed within 6 hours after admission. The patients were followed up for the development of pancreatitis complications both during their hospitalization and up to 4-6 weeks after discharge. Twenty-one healthy volunteers who presented to the gastroenterology outpatient clinic with dyspepsia and did not have any chronic disease were used as the control group. The control group was required to have no febrile illness (including coronavirus disease 2019) in the last 2 weeks and to have normal hemogram and basic biochemical tests. Patients younger than 18 years of age and patients with inflammatory bowel disease, celiac disease, all malignancies, chronic renal failure, diabetes, psychiatric disorder, or pregnancy were excluded from the study.

### Data Collection

Age and gender of all participants were recorded. The etiology of AP, length of hospital stay, and in-hospital mortality rate were determined. In addition, the development of organ dysfunction, complications of pancreatitis, and sepsis were monitored and recorded. Organ dysfunction and sepsis were chosen as outcome parameters. The severity of pancreatitis and its complications such as peripancreatic fluid collection, necrosis, and infected necrosis were determined according to the 2012 revision of the International Atlanta Symposium on Acute Pancreatitis guidelines.^4^ Arrival and 24-hour APACHE II score,^15^ the 48-hour Ranson score,^16^ the Bedside Index for Severity in Acute Pancreatitis (BISAP) score,^17^ and CRP (C-reactive protein) values at 48 hours^18^ were calculated to estimate disease severity.

### Biochemical Analyses

The blood samples were taken at the time of the diagnosis of pancreatitis in the emergency department. Then, they were centrifuged (Nüve, NF-800 Serial no.04.4136, Turkey) at 3000 × ***g*** for 10 minutes, and the sera were stored at –20˚C. On the evaluation day, the samples were melted at room temperature.

The concentrations of human zonulin in sera were measured using commercially available human zonulin ELISA (Enzyme Linked Immunosorbent Assay) Kit (BT Lab Company, Cat No. E3704Hu, Zhejiang, China). The enzymatic reactions were quantified in an automatic microplate photometer. The concentrations of zonulin were determined by comparing the optic density of the samples to the standard curve. The mean interassay and intra-assay coefficients of variation percentage for zonulin were <10% and <8%, respectively. The assay range of the kit was 0.3-90 ng/mL. The sensitivity of test was 0.13 ng/mL. All assays were conducted according to the instructions of the manufacturer. The samples, which showed higher concentrations, were diluted and measured in duplicate.

### Data Analysis

In descriptive statistics, mean, SD, median value, and 25%-75% values were used for numerical variables, and numbers and percentages were used for categorical variables. The independent samples *t*-test was used to analyze differences between 2 groups of variables with parametric distribution. The Mann–Whitney *U*-test was used to analyze differences between 2 groups of variables with non-parametric distribution, and the Kruskal–Wallis test was used to analyze differences between more than 2 groups of variables with non-parametric distribution. Pearson’s Chi-square test was used for intergroup comparisons of categorical variables. When a significant difference was detected in comparisons between more than 2 groups, post-hoc analysis was performed to understand from which group the difference originated. The CI was set as 95%, and the significance level was accepted as *P* < .05. Power analysis was done with nQuery advisor version 7. Statistical analysis was carried out using the Statistical Package for Social Sciences Version 22.

## Results

### Patient Characteristics

A total of 58 patients (35 women, 23 men, median age: 55.8 years) and 21 healthy volunteers (12 women, 9 men, median age: 51.9 years) were included in our study. There was no significant difference in age and gender between the patient and control groups and the subgroups according to the severity of pancreatitis. Disease severity was mild in 46 (79%) patients, moderately severe in 7 (12%) patients, and severe in 5 (9%) patients. Although the age was higher in patients with pancreatitis, the mean age of the acute severe pancreatitis group was lower and the rate of female patients was higher. The most common cause of pancreatitis was gallstones, and the second was pancreatitis after endoscopic retrograde cholangiopancreatography. In-hospital mortality was not observed in any patient. The length of stay was significantly longer in the severe pancreatitis group than in the mild pancreatitis group. A total of 12 (20%) patients had AP complications. Seven of the complications were necrosis (12%) (3 infected necrosis and 4 peripancreatic or pancreatic necrosis) and 5 were peripancreatic fluid collection (8%). While sepsis was not seen in mild cases, 1 (14.2%) of moderately severe cases and 2 (40%) of severe cases had sepsis. Arrival APACHE score, Ranson score (48th hour), BISAP score, and CRP level were statistically significantly higher in cases with severe pancreatitis. The demographic characteristics of patients with AP and the control group, the clinical status of patients with AP according to the revised Atlanta criteria, and the serum zonulin levels of both groups are given in Table 1.

### Zonulin Levels

**Figure 1.**
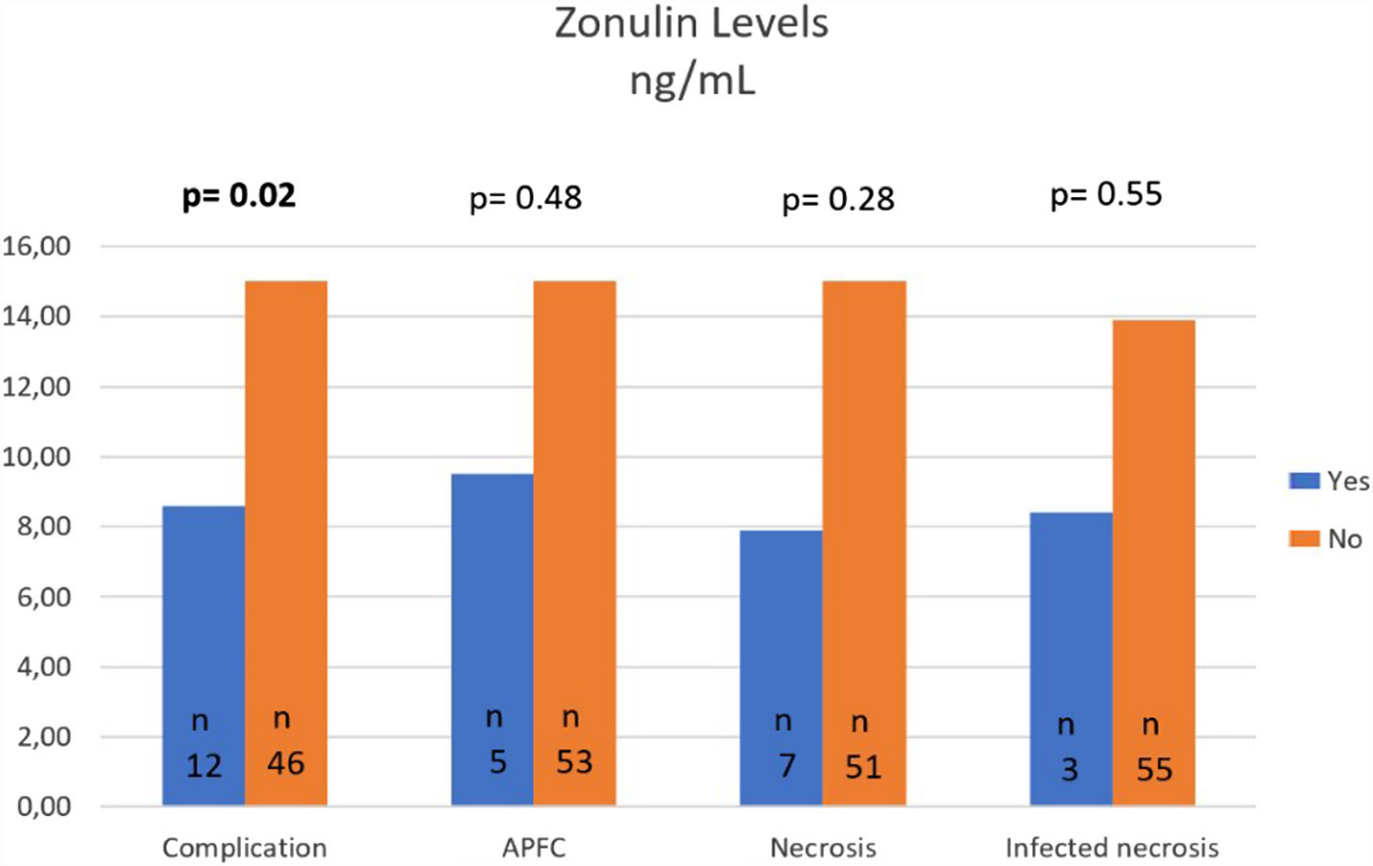
The relationship of zonulin levels with the complications of acute pancreatitis.

Zonulin levels were high in the control group, whereas it had the lowest mean in the severe pancreatitis group (Table 1). No significant difference was observed when zonulin levels were compared according to disease severity (Table 2). There was no significant difference between zonulin levels in patients who developed organ dysfunction or sepsis compared to those who did not (Table 3). In 12 patients who had AP complication, zonulin levels were found to be significantly lower (*P* < .02), with a mean of 8.6 (Figure 1). When subgroup analysis was performed in terms of peripancreatic fluid collection, necrosis, and infected necrosis, no significant difference was found between the groups (Figure 1). Zonulin levels were not significantly correlated with different parameters (Table 4).

## Discussion

Our study is the first clinical study in the literature to evaluate zonulin as an early biomarker in the development of disease severity and complications in AP. Although the results show us that zonulin is not a good biomarker in indicating the severity of AP, low zonulin levels may be an early indicator of the development of complications. Currently, the problem in clinical practice in AP is not being able to predict which patient will develop complications and which patient will have a more severe course rather than diagnosis. Disease severity and mortality in AP are closely related to the development of pancreatic necrosis and necrosis infection, the main source of which is thought to be the intestinal tract.^8^ Bacterial translocation and intestinal barrier dysfunction play an important role in the development of this process; moreover, it can lead to sepsis and organ dysfunction. Maintaining the intestinal barrier function is of great importance in preventing the development of acute necrotizing pancreatitis.^19^ This protein is secreted from the intestines, liver, and many other organs and binds its receptors on the ileum and jejunum. By binding these receptors, tight junctions between cells are regulated, and thus the permeability of the small intestine increases.^20,21^ Zonulin has been associated with a variety of clinical conditions. These include celiac disease, type 1 diabetes, fatty liver, various cancers, and rheumatoid arthritis.^10^ Wang et al^13^ has recently reported that zonulin has a very important role in the formation of intestinal immunity. It has been shown that there is a decrease in the barrier function of the intestines and an increase in bacterial translocation at the onset of AP.^6,7^ It has been determined that bacterial translocation develops with the deterioration of intestinal barrier function due to AP, leading to systemic inflammation and sepsis.^8^ It has been proven that damage to tight and adherens junctions by various factors plays a role in the development of intestinal barrier dysfunction in AP.^9^ From this point of view, we aimed to show whether there was a change in serum zonulin levels in patients with AP. In our study, zonulin levels were found to be low in the pancreatitis group, in those with severe pancreatitis, and in those who developed sepsis and organ dysfunction, without reaching a statistical significance. There was no correlation between pancreatitis severity and zonulin levels. It was found to be significantly lower only in the group who developed complication of pancreatitis. In a recent study by Yıldız et al.^19^ zonulin levels were found to be high in intestinal tissue and serum of rats with experimentally induced acute severe pancreatitis. In our study, zonulin levels were lower in AP or severe pancreatitis compared to the control group, but they were statistically insignificant. In addition, although zonulin levels were found to be low in the group that developed infected necrosis, which was known as the most related complication of bacterial translocation, no significant difference was observed. The difference between the 2 studies may be due to different pathophysiological processes in rats and humans. In an experimental study conducted by Huang et al.^8^ no significant difference was found in the levels of ZO-1, a tight junction protein, in the acute necrotizing pancreatitis group.^8^ In another study, it was shown that occludin and ZO-1 expression in the intestinal tissue decreased in experimental acute severe pancreatitis.^22^ Sonika et al^23^ demonstrated that the intestinal permeability increased in patients with AP. Decreased expression of claudin 4 protein in duodenal biopsies was blamed for this increase. However, a tight junction protein that could be measured in serum was not examined in the study. It is clear that intestinal permeability is increased in AP, but there seems to be no consensus in the literature regarding the change of tight junction proteins and the mechanism of their role in showing the severity of the disease. This may also be due to the current lack of sufficient clinical studies. In addition, there are recent publications in the literature that commercial zonulin ELISA kits do not reflect intestinal permeability.^24^ Although the inadequacy of zonulin in demonstrating the development of infected necrosis seems to be associated with this, the evaluation by Alessio Fasano shows that the measurement of the zonulin family of proteins is still a good indicator of intestinal permeability.^25^ According to our results, which are the first in the literature, low zonulin levels in patients with AP may be associated with the development of pancreatic complications. This may be a guide for patients with pancreatitis who may need early and aggressive resuscitation.

The main limitation of our study was the small number of patients. Studies with a larger number of participants might affect the result. In addition, mortality analysis could not be performed since there was no in-hospital mortality in our study.

## Conclusion

Our results showed that zonulin levels are not a guide in the diagnosis of AP, in determining its severity, and in the development of sepsis and organ dysfunction. However, it can be speculated that it may be a negative marker in the early stages of pancreatitis in demonstrating the occurrence of pancreatitis complications. Our study is valuable because it is the first clinical study investigating the relationship between AP and zonulin levels. Future prospective studies involving more patients will guide to clarify the uncertain points on this subject.

## Figures and Tables

**Table 1.** Clinical Features and Demographic Data of Patients and Control Group

	Clinical Status of Patients with Acute Pancreatitis According to 2012 Revised Atlanta Classification					
	Mild, n = 46	Moderately Severe, n = 7	*P*	Severe, n = 5	*P* ^*^	Control, n = 21
Age	55.5 (18-87)	60.7 (46-78)	.47	51.4 (32-79)	.63	51.9 (28-78)
Sex, male/female	21/25	2/5	–	0/5	–	9/12
Etiology, n (%)						
Biliary	35/46 (76)	2/7 (28)		4/5 (80)		
Post-ERCP	7/46 (15.2)	2/7 (28)		1/5 (20)		
Hyperlipidemia	2/46 (4.3)	0/7 (0)		0/5 (0)		
Alcohol	0/46 (0)	1/7 (14)		0/5 (0)		
Other	2/46 (4.3)	2/7 (28)		0/5 (0)		
In-hospital mortality	0	0	–	0	–	
Length of stay	5.8 (3-22)	12.8 (7-22)	.04	17.6 (9-23)	.008	
Organ dysfunction, n, (%)	0 (0)	2 (28)		5 (100)		
Pulmonary	0/46 (0)	1/7 (14.2)		4/5 (80)		
Hepatic	0/46 (0)	1/7 (14.2)		1/5 (20)		
Renal	0/46 (0)	1/7 (14.2)		1/5 (20)		
Sepsis, n (%)	0/46 (0)	1/7 (14.2)		2/5 (40)		
APACHE arrival	9 (3-15)	11 (3-16)	.27	11 (7-17)	.38	
APACHE (24th hour)	9 (3-15)	10 (3-16)	.42	11 (7-16)	.37	
Ranson arrival	1.1 (0-3)	1.4 (0-3)	.65	1.8 (1-3)	.1	
Ranson (48th hour)	2.3 (0-3)	2.7 (0-3)	.69	2.2 (1-3)	.05	
BISAP score	0.5 (0-2)	1.1 (0-4)	.03	2.5 (1-5)	<.00	
CRP (48th hour)	68.3 (4-402)	130.7 (33-308)	.16	241.4 (187-297)	<.00	
Zonulin, ng/mL	13.7 (5.1-87.2)	17.2 (6.3-68.1)	.7	7.8 (5.8-9.7)	.38	23.1 (4.3-85.7)

^*^ *P* value is for mild versus severe acute pancreatitis.

BISAP, Bedside Index for Severity in Acute Pancreatitis; CRP, C-reactive protein; ERCP, endoscopic retrograde cholangiopancreatography.

**Table 2.** Zonulin Levels According to Patient–Control and Pancreatitis Severity

Zonulin, ng/mL	Acute Pancreatitis, n = 58	Control, n = 21	*P*
	13.6 ± 15.4	23.1 ± 23	.09
	Mild AP, n = 46	Moderately severe AP, n = 7	*P*
	13.7 ± 15	17.2 ± 22.5	.5
	Mild AP, n = 46	Severe AP, n = 5	*P*
	13.7 ± 15	7.8 ± 1.4	.38
	Moderately severe AP, n = 7	Severe AP, n = 5	*P*
	17.2 ± 22.5	7.8 ±1.4	.37

AP; acute pancreatitis.

**Table 3.** The Relationship of Zonulin Levels with the Development of Organ Dysfunction and Sepsis

Zonulin, ng/mL	Organ Dysfunction		*P*
	Yes n = 7	No n = 51	
	8.4 ± 2.6	14.4 ± 16.3	.34
	Sepsis		
	Yes n = 4	No n = 54	
	8.9 ± 3.6	14 ± 15.9	0.52

**Table 4.** Correlation Analysis of Zonulin and Different Parameters (Non-parametric Spearman)

Correlation	*r*	*P*
APACHE, arrival	–0.109	.41
APACHE (24th hour)	–0.09	.46
Ranson, arrival	0.005	.9
Ranson (48th hour)	–0.12	.93
BISAP score	–0.83	.5
CRP (48th hour)	0.53	.6

BISAP, Bedside Index for Severity in Acute Pancreatitis; CRP, C-reactive protein.
